# A dwarf walrus from the Miocene of Baja California Sur, Mexico

**DOI:** 10.1098/rsos.180423

**Published:** 2018-08-08

**Authors:** Jorge Velez-Juarbe, Fernando M. Salinas-Márquez

**Affiliations:** 1 Department of Mammalogy, Natural History Museum of Los Angeles County, 900 Exposition Boulevard, Los Angeles, CA 90007, USA; 2 Department of Paleobiology, National Museum of Natural History, Smithsonian Institution, Washington, DC 20560, USA; 3 CIENTIFICA MENTE, Calle 15, Número 220, Int. 7, Zona Centro, Ensenada, Baja California 22800, Mexico

**Keywords:** Odobenidae, marine mammals, eastern Pacific, Miocene

## Abstract

Here, we describe the odobenid *Nanodobenus arandai* gen. et sp. nov., based on a nearly complete left mandible from the mid to late Miocene Tortugas Formation in Baja California Sur. *Nanodobenus* is distinguished among odobenids by displaying a unique combination of plesiomorphic and derived characters, such as narrow mandibular symphysis, well-developed genial tuberosity, bilobed canine and p2 roots, bulbous post-canine teeth with the paraconid, protoconid and hypoconid, and smooth lingual cingula. Moreover, it is characterized by its small adult body length, which is estimated at about 1.65 m. Throughout the Miocene–Pliocene odobenids are characterized by an increase in body size, especially after the extinction of desmatophocids in the late Miocene. The small size of *Nanodobenus* departs from this trend, demonstrating that there was greater size disparity among odobenids in the mid–late Miocene than previously thought. It is hypothesized that *Nanodobenus* occupied a niche that was later on occupied by similar-sized otariids, such as *Thalassoleon mexicanus,* which occurs sympatrically with large odobenids in the overlying Almejas Formation.

## 1. Introduction

The evolutionary history of odobenids is intimately tied with the North Pacific, with the group originating in the region during the early Miocene [1,2]. As such, their fossil record offers unique insight into their ancient diversity and evolutionary patterns over time [1–4]. During their early history odobenids occurred sympatrically with otariids and desmatophocids, with the latter having larger body sizes and presumably occupying higher trophic levels [1–6]. This pattern shifted after the mid–late Miocene extinction of desmatophocids, leading to a greater diversity of odobenids and an overall increase in body size within this group, peaking during the latest Miocene–early Pliocene [3,4]. These multispecies communities were then composed of otariids and usually two or more species of large (greater than or equal to 2.5 m in body length) odobenids [3,4].

Here, we describe a new odobenid from mid–late Miocene deposits in Baja California Sur, Mexico. The new taxon is characterized by its small body size, representing a case of dwarfism in a group that is otherwise characterized by an increase in body size over time [3,7].

## 2. Material and methods

### 2.1. Phylogenetic analysis

For the phylogenetic analysis, we used the matrix of Tanaka & Kohno [8] as modified by Velez-Juarbe [9], by adding UABC FCM 0072 (see electronic supplementary material). All characters were equally weighted and unordered. A Bayesian inference analysis was performed using MrBayes [10] using the following parameters: mcmc = 3 000 000, saplefreq = 1000, printfreq = 1000, starttree = random. The heuristic analysis was performed using PAUP* [11] by doing a heuristic search with 1000 replicas; statistical support was obtained by doing 1000 bootstrap replicas.

### 2.2. Institutional abbreviations

*LACM*, Departments of Vertebrate Paleontology and Mammalogy, Natural History Museum of Los Angeles County, California, USA; *LACM Loc.*, Vertebrate Paleontology Locality, Natural History Museum of Los Angeles County, California, USA; *LC*, Ralph B. Clark (formerly ‘Los Coyotes’) Regional Park, Interpretive Center, Buena Park, California, USA; *MNHN.F.SAS*, Muséum national d'Histoire naturelle, Paris, France; *OCPC*, Orange County Paleontological Collection, John D. Cooper Archaeological and Paleontological Center, Santa Ana, California, USA; *SDNHM*, San Diego Natural History Museum, San Diego, California, USA; *SFMV*, Shiga Fossil Museum, Vertebrate Collection, Matsumoto, Nagano, Japan; *UABC FCM*, Universidad Autónoma de Baja California, Facultad de Ciencias Marinas, Baja California, Mexico; *UCMP*, University of California Museum of Paleontology, Berkeley, California, USA; *USNM*, Department of Paleobiology, National Museum of Natural History, Washington, DC, USA.

### 2.3. Specimens observed

*Aivukus cedrosensis* (LACM 154671, cast of type); *Allodesmus kernensis* (LACM 4320, 138167, 152730); *Atopotarus courseni* (LACM 1376); *Callorhinus ursinus* (LACM 51353, 51354, 51357, 52342, 52398); *Desmatophoca* sp. (LACM 123811, 123815, 159024, 159025); *Desmatophoca brachycephala* (LACM 120199); *Dusignathus santacruzensis* (LACM 1527, cast of type; LACM 3011, 4342); *Dusignathus seftoni* (LACM 155310, cast of type; LACM 135545, cast of SDNHM 20801); *Eotaria citrica* (LACM 122666); *Eotaria crypta* (LACM 159981; OCPC 5710); *Gomphotaria pugnax* (LACM 105151, 121508; LC 7750); *Hadrokirus martini* (MNHN.F.SAS 1627); *Imagotaria* sp. (LACM 45837, cast of UCMP 85197); *Imagotaria downsi* (LACM 144453, cast of type; USNM 23858, 184060); *Neomonachus schauinslandi* (LACM 543854); *Neotherium mirum* (LACM 123000, 123002, 127697; UCMP 81665); Odobenidae gen. et sp. indet (LACM 135920); *Odobenus rosmarus* (LACM 31336, 52376, 52423, 72561); *Ontocetus* sp. (LACM 150001, cast of SFMCV-0001); *Pelagiarctos* sp. (SDNHM 131041); *Pelagiarctos thomasi* (LACM 121501); *Pithanotaria starri* (LACM 115153, 115677); *Proneotherium* sp. (LACM 128412); *Protodobenus japonicus* (LACM 140726, cast of type); *Prototaria planicephala* (LACM 134826, cast of type); *Prototaria primigena* (LACM 130432, cast of type).

## 3. Systematic palaeontology

**Pinnipedia** Illiger, 1811

**Odobenidae** Allen, 1880

***Nanodobenus arandai****,* gen. et sp. nov.

**LSID**: zoobank.org:act:47A6AC56-2173-4380-920F-B95E3ED197A0

**Etymology.** The name derives from the combination of ‘*nano*’, from the Latin ‘*nanus*’ which translates to dwarf, in reference to the small size of the specimen and estimated body size, combined with *Odobenus*, the genus of extant walrus, *Odobenus rosmarus* (Linnaeus, 1758), and the occasionally used suffix in odobenids (e.g. *Archaeodobenus*). The specific epithet honours Dr Francisco Aranda-Manteca (UABC) in recognition of his mentorship to the junior author and contributions to the knowledge of extinct marine vertebrates of Baja California and Baja California Sur.

**Holotype.** UABC FCM 0072, nearly complete left mandible, including p2–4. Collected by T. McMillan, c. 1987.

**Figure 1.**
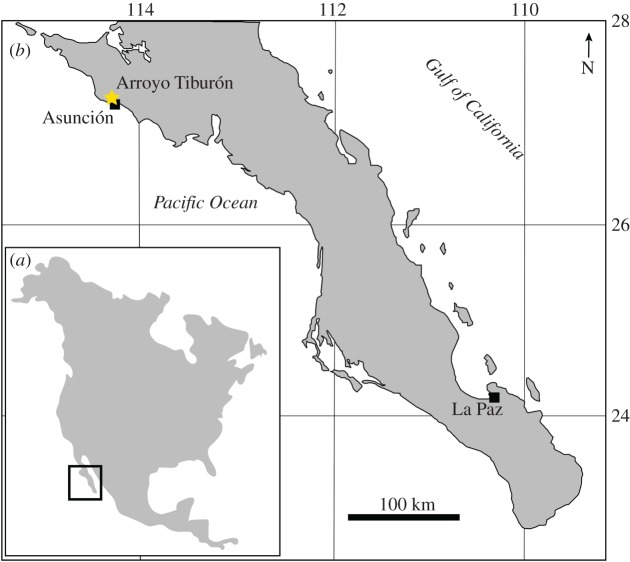
Map of North America (*a*) showing the general study area, and map of Baja California Sur (*b*) showing the type locality at Arroyo Tiburón.

**Type locality.** Arroyo La Chiva (=Arroyo Tiburón [12–14]), Asunción, Baja California Sur, Mexico (figure 1).

**Formation and age.** The geology around the type locality was mapped by Throughton [12] who referred to the sediments exposed here as the Tortugas Formation. The age of the Tortugas Fm. seems to be relatively broad across its distribution, ranging over mid–late Miocene [15]. The exposure at Arroyo La Chiva (Arroyo Tiburón) was dated by Pérez-Guzmán [16] to between 15.7 and 7.8 Ma, based on the occurrence of radiolarians of the *Dorcadospyris alata* and *Diartus hughesi* zones [17]. Moreno-Ruiz & Carreño [14] studied the diatomites below the overlying Almejas Fm. in this area, and report diatoms belonging to the *Denticulopsis hustedtii*-*D.* lauta Subzone D, which gives it an age between 9.9 and 9.2 Ma [18]. The bone-bearing units are towards the lower part of the formation [12,13], therefore we restrict the range for UABC FCM 0072 and LACM 60914 to between 15.7 and 9.2 Ma (Langhian–mid Tortonian: mid to late Miocene). Througton [12] and Barnes [15] provided lists of marine mammals from the type locality, including desmostylians, dugongids, odontocetes, mysticetes, otariids, odobenids and desmatophocids; however, they did not provide specimen numbers. Examination of material housed at LACM by the senior author yielded the referred specimen (see below), and confirmed the presence of Mysticeti (60912), Odontoceti (60911), Desmostylia (LACM 60919), Paleoparadoxiidae (LACM 60918) and Otariidae (LACM 60915); identification of material in other collections still needs to be revised.

**Tentatively referred specimen.** LACM 60914, right calcaneum, collected by G. H. Throughton, 11 March 1974. From locality LACM 3892, Arroyo La Chiva (Arroyo Tiburón [12–14]), Asunción, Baja California Sur, Mexico.

**Range.** Mid–late Miocene of Baja California Sur, Mexico.

**Differential diagnosis.** Identified as an odobenid based on the presence of post-canine teeth that are bulbous, longer than high and with smooth enamel. Smallest known odobenid, with a body length estimated at 1.65 m (based on [7]; table 3); further characterized by the following unique combination of mandibular characters: mandibular symphysis with a subtriangular outline (narrower posteroventrally), symphysis short (shared with *Proneotherium repenningi*, *Neotherium mirum*, *Kamtschatarctos sinelnikovae* and *Pontolis magnus*), genial tuberosity extending well below ventral margin of ramus (shared with *Pelagiarctos* spp., *Archaeodobenus akamatsui*, *Imagotaria downsi*, *P. magnus*, *Dusignathus* spp. and *Ontocetus emmonsi*), and located posterior to p2 (shared with *P. magnus*, *Dusignathus* spp., *Gomphotaria pugnax* and *O. emmonsi*). Differing further by the following dental characters: lower canine with bilobed root (shared with *N. mirum*, *Pelagiarctos* spp. and *I. downsi*); well-developed enamel on post-canine teeth (as in most early odobenids, and differing from *P. magnus*, *Dusignathus* spp., *G. pugnax* and odobenines); post-canine teeth with small, but distinct paraconid cusp (shared with *N. mirum*, *K. sinelnikovae*, *I. downsi*, *Pelagiarctos* spp. and *A. akamatsui*); absence of a talonid basin on the post-canine teeth (shared with *Pr. repenningi*, *A. akamatsui* and odobenines), p2 root bilobed, double rooted p3–4 and retention of m2 (differing from the single roots and lack of m2 of *Dusignathus* spp., *G. pugnax* and more derived odobenids).

**Figure 2.**
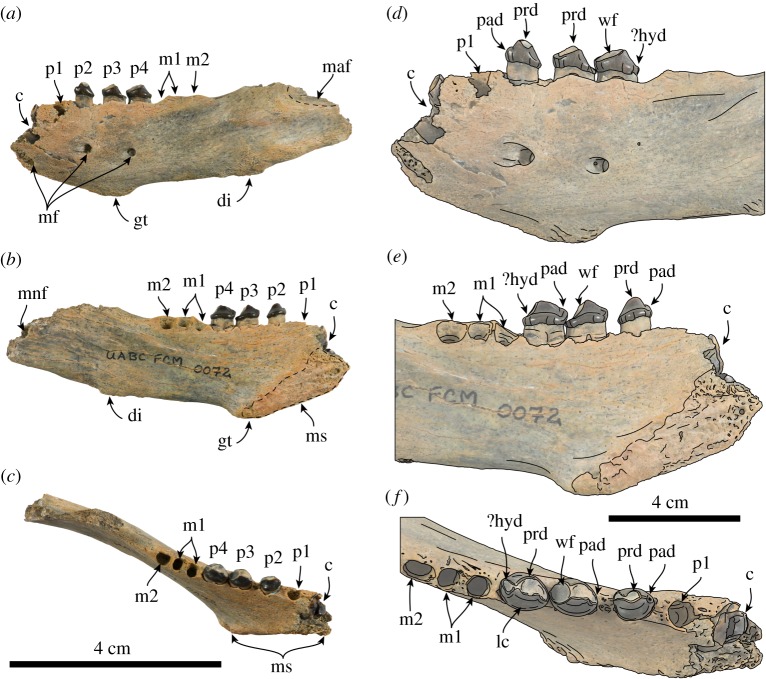
Mandible and lower dentition of *Nanodobenus arandai* gen. et sp. nov. (UABC FCM 0072). Mandible in lateral (*a*), medial (*b*) and occlusal (*c*) views. Lower dentition in labial (*d*), lingual (*e*) and occlusal (*f*) views. Abbreviations: c, lower canine; di, digastric insertion; gt, genial tuberosity; hyd, hypoconid; lc, lingual cingulum; p1–4, lower premolars 1–4; m1–2, lower molars 1–2; maf, masseteric fossa; mf, mental foramina; mnf, mandibular foramen; ms, mandibular sumphysis; pad, paraconid; prd, protoconid; wf, wear facet.

**Description and comparison of mandible.** The holotype mandible represents an adult individual based on the fully erupted dentition, worn crowns and rough symphyseal surface. The mandibular symphysis is unfused as in most odobenids, with the exception of *Pelagiarctos thomasi*, *Dusignathus seftoni*, *Valenictus chulavistensis* and *Odobenus rosmarus* [6,19–21]. The symphysis has a low angle (approx. 35°) relative to the dorsal edge of the mandible and is anteroposteriorly short (table 1); the surface has a triangular outline and the surface is marked by pits and ridges (figure 2), reminiscent of the symphysis of *Imagotaria* sp. from the Santa Margarita Fm. (=Desmatophocine A [4,22]). The posteroventral end of the symphysis forms the genial tuberosity, which extends far below the ventral border of the horizontal ramus and is the dorsoventrally deepest part of the mandible (figure 2). The genial tuberosity is located far posteriorly at a point ventral to p3, differing from most odobenids (e.g. *Neotherium mirum*, *Imagotaria downsi* [5,9,23]). The anterior surface of the symphysis is smooth, with a single rounded anterior mental foramen (2.7 mm in diameter); two other mental foramina are along the lateral surface of the mandible, below p2–4, and have similar diameter (2.8–3 mm). In the dorsal view the ramus forms a nearly parallel mandibular arch as in most early odobenids. The dorsal and ventral edges of the horizontal ramus are nearly parallel anteriorly, but posteroventrally is interrupted by a raised irregularly oval, rough digastric insertion, giving it a more sinuous outline. The digastric insertion is prominent, but not to the degree seen in *Pontolis magnus*, *Dusignathus* spp. or desmatophocids, being more similar to *Imagotaria downsi* [4,5,19,21]. The anteroventral edge of the masseteric fossa is preserved and seems to have been relatively deep.

**Table 1.** Measurements (in millimetres) of mandible of *Nanodobenus arandai* gen. et sp. nov. (UABC FCM 0072) (modified from Velez-Juarbe [3]).

maximum length as preserved	105.08
anterodorsal–posteroventral height of symphysis	33.92
anteroventral–posterodorsal length of symphysis	16.48
symphyseal angle	35°
height at genial tuberosity	30.06
mandible height/width at p1	25.54/9.52
mandible height/width at p2	27.34/8.62
mandible height/width at p3	29.20/8.46
mandible height/width at p4	25.68/7.54
mandible height/width at m1	26.3/7.58
mandible height/width at m2	25.72/7.24
length of tooth row c1-m2	56.54
length of post-canine tooth row	46.74
diastema between c and p1	3.50
diastema between p1 and 2	2.34
diastema between p2 and 3	2.54
diastema between p3 and 4	—
diastema between p4 and m1	0.78
diastema between m1 and 2	0.60
canine: transverse width/anteroposterior length	7.58/10.42
p1: transverse width/anteroposterior length of alveolus	7.58/10.42
p2: transverse width/anteroposterior length/height	5.02/6.66/4.54
p3: transverse width/anteroposterior length/height	5.74/8.34/5.20
p4: transverse width/anteroposterior length/height	5.78/8.06/4.92
m1: transverse width/anteroposterior length of alveolus	3.22/8.86
m2: transverse width/anteroposterior length of alveolus	3.54/5.08

The number of incisors is unknown. The canine is broken at the base and the root has a labial longitudinal sulcus as in *Neotherium mirum*, *Pelagiarctos thomasi*, *Imagotaria downsi* and *Imagotaria* sp. [6,9,22,23]. The first premolar is represented by a rounded, single alveolus that is about the same diameter as the other post-canine teeth; p2 is bilobed, while p3–m1 are double rooted and m2 has a single rounded alveolus (figure 2). The crowns of p2–4 are bulbous, and longer than high, with smooth, nearly straight lingual cingula that curves anterodorsally towards the paraconid, somewhat resembling the condition seen in some specimens of *Imagotaria downsi* (e.g. USNM 23858), and unlike the dorsally arched cingula of *Neotherium mirum* [5,9]. The crowns are worn apically, and along their distal and mesial edges, they are dominated by the protoconid, and have a small paraconid on p2, and, although worn, seemed to have had a relatively well-developed paraconid on p3–4; the distal margin of p2–4 is worn, but a hypoconid seems to have been present.

Affinities of UABC FCM 0072 with pinniped groups other than odobenids can be ruled out based on a number of characteristics. The post-canine teeth of *Nanodobenus* are bulbous and longer than high, in contrast to the transversely narrow, sharply triangular teeth generally seen in otariids (e.g. *Pithanotaria starri* [3]) or the bulbous, higher than long, or subequal post-canines of desmatophocids (e.g. *Desmatophoca oregonensis* [24], *Allodesmus kernensis* (LACM 138167)). Some phocids also have bulbous, longer than high post-canine teeth, but the enamel surface is usually carinated (e.g. *Hadrokirus martini* [25], *Neomonachus* spp.), unlike the smooth enamel of UABC FCM 0072. *Nanodobenus* differs further from desmatophocids by lacking the greatly expanded digastric insertion [4,24]. Furthermore, the lower canine of *Nanodobenus* has a bilobed root, which is a characteristic it shares with other odobenids and that seems to be absent in other pinniped groups.

**Figure 3.**
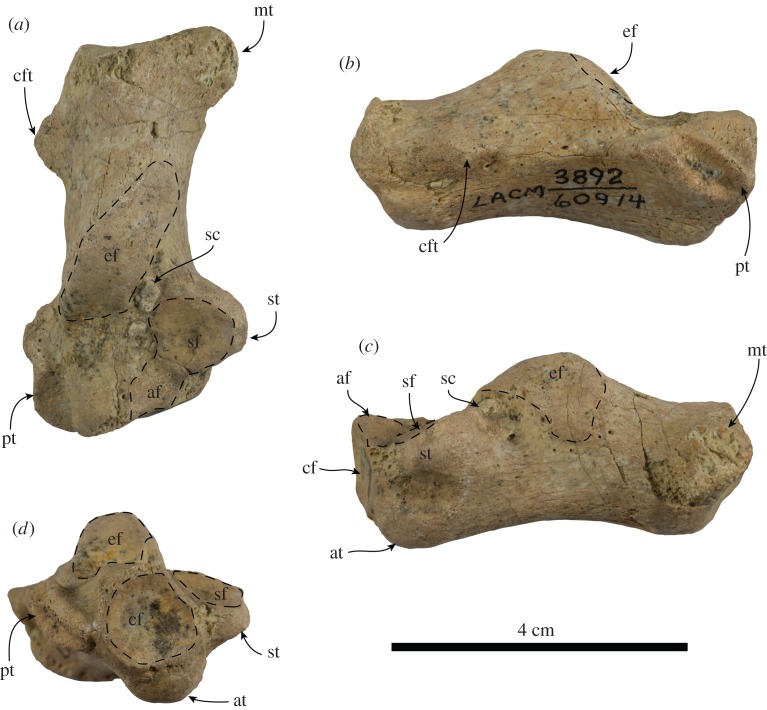
Right calcaneum (LACM 60914) referred to *Nanodobenus arandai* gen. et sp. nov., in dorsal (*a*), lateral (*b*), medial (*c*) and distal (*d*) views. Abbreviations: at, anterior tubercle; af, accessory facet; cf, cuboid facet; cft, calcaneofibulare tuberosity; ef, ectal facet; mt, medial tuberosity; pt, peroneal tubercle; sc, sulcus calcaneus; sf, sustentacular facetl; st, sustentaculum tali.

**Referred calcaneum.** The calcaneum is completely preserved (figure 3). The calcaneal tuber is elonganted with a prominent medial tuberosity, similar to the condition observed in odobenids (e.g. *Neotherium mirum*, *Imagotaria downsi*, *Proneotherium repenningi* [5,20,26,27]). The posterior surface of the calcaneal tuber is transversely concave with a nearly rounded outline. Proximally, just distal to the posterolateral corner of the calcaneal tuber is a prominent, subconical rugosity (herein termed calcaneofibulare tuberosity), probably marking the attachment of the calcaneofibulare ligament; a similarly positioned, but much lower rugosity is also present in *Proneotherium repenningi* and observed specimens of *Neotherium mirum* (LACM 51077 and LACM 127997) [26,27]. Dorsally, the ectal facet is elongated, broadly convex, with its posterior half oriented medially, while its distal half faces anterodorsally, similar to that of *Neotherium mirum* and *Proneotherium repenningi* [26,27]. Distomedial to the ectal facet, the sustentaculum tali projects as far medially as the medial tuberosity; the sustentacular facet faces dorsally and slightly anteriorly, forming a shallowly concave, rounded surface. As in *Proneotherium repenningi*, the sustentacular facet is continuous distally with an accessory facet that reaches the dorsomedial corner of the cuboid facet [27]. The sustentacular facet and the ectal facet are divided by a relatively shallow sulcus calcanei. Distolaterally the peroneal tubercle forms an elongated shelf; it has a shallow sulcus on its dorsal surface and another one that extends obliquely along its lateral surface. Anteriorly, the cuboid facet is shallowly concave and has a round outline; it forms an angle of approximately 70° relative to the long axis of the bone. Ventromedial to the cuboid facet is a prominent, knob-like, anterior tubercle.

We are confident that LACM 60914 represents an odobenid, as it has a prominent medial tuberosity, which can be considered as an odobenid synapomorphy [5,20]. The overall morphology of LACM 60914 is similar to that of *Neotherium mirum*, differing mainly, from this and other odobenids in the enlarged attachment of the calcaneofibulare ligament. The Tortugas calcaneum is close in size to the smallest calcaneum of *Neotherium mirum* examined (LACM 127997; table 2) and to the lectotype of *Neotherium mirum*, and notably smaller than that of *Proneotherium repenningi* [26–28]. Size differences among specimens of *Neotherium mirum* are thought to represent sexual dimorphism; with female individuals being smaller than their male counterparts, it is possible that LACM 60914 represents a male individual of *Nanodobenus* [5,6,9]. However, because multiple pinnipeds, including more than one odobenid, are supposed to be present in the Tortugas Fm. [15], and lack of overlap with the type, we tentatively assign LACM 60914 to *Nanodobenus arandai*.

**Table 2.** Measurement of calcanei of *Nanodobenus arandai* gen. et sp. nov. (LACM 60914) from the Tortugas Fm. and *Neotherium mirum* (LACM 21254, 51077, 127997) from the Sharktooth Hill Bonebed (modified from Kellogg [26]).

	LACM 60914	LACM 21254	LACM 51077	LACM 127997
maximum length	52.46	63.32	68.28	52.54
maximum width proximally	31.92	34.40	31.14	21.60
maximum height proximally	21.26	25.14	24.20	17.00
maximum width distally	23.10	28.60	39.38	26.84
maximum height distally	18.36	22.98	25.84	20.46
maximum length of the ectal facet	24.42	25.84	26.34	20.80

## 4. Results

**Figure 4.**
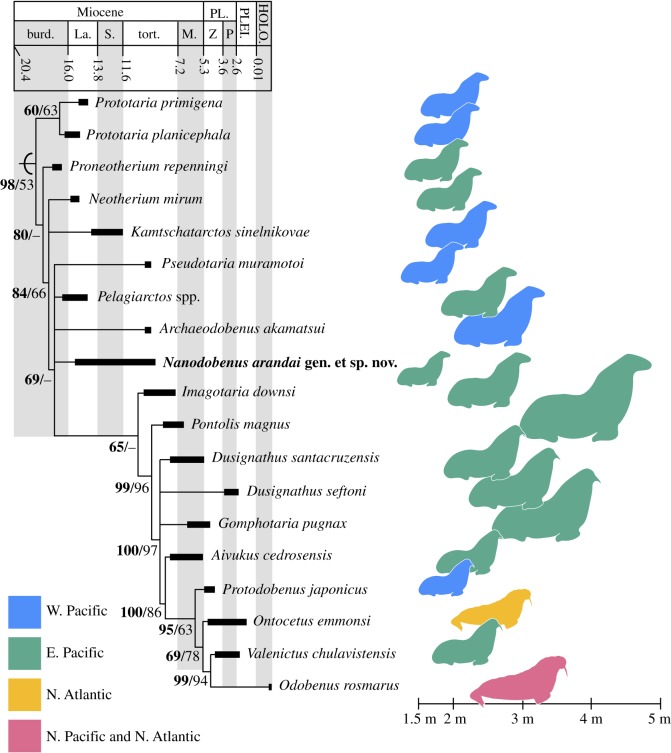
Time-calibrated strict consensus tree of Odobenidae and body size distribution. Species range from [3,4,29]; body size estimates from table 3 and outlines modified from Berta *et al*. [2] and Lydersen [30]. Numbers in nodes represent posterior probability (in bold) and bootstrap values.

The Bayesian inference tree and the consensus tree obtained from the heuristic search (336 most parsimonious trees, 300 steps long, with ci = 0.493 and ri = 0.724) had very similar topologies, with the former having better resolution among the more derived taxa (figure 4). The topology closely resembles that of recent works on odobenids [8,9,21]. *Nanodobenus* falls in a polytomy with *Pseudotaria muramotoi*, *Pelagiarctos* spp. and *Archaedobenus akamatsui*. The lack of resolution is probably due to incompletely known taxa such as *Pelagiarctos* spp. and *Nanodobenus*, which can only be scored for 35% and 28% of the characters; this was part of the reason *Pelagiarctos* was not included in some analyses [8]. Nevertheless, *Nanodobenus arandai* shares unique features with basal and derived odobenids as described above. Reduction/fusion of the post-canine root lobes happened multiple times in odobenids and in this respect *Nanodobenus* resembles some specimens that have been referred to as *Imagotaria* spp. [4,5,9,22].

**Table 3.** Odobenid body length estimates used in the phylogenetic analysis.

species	body length (cm)	source
*Prototaria primigenia*	211	[7]
*Prototaria planicephala*	202	[7]
*Proneotherium repenningi*	198	[7]
*Neotherium mirum*	202	[7]
*Kamtschatarctos sinelnikovae*	221	[7]
*Pseudotaria muramotoi*	194	[7]
*Pelagiarctos* spp.	234	[7]
*Archaeodobenus akamatsui*	280	[8]
*Imagotaria downsi*	257	[7]
*Nanodobenus arandai*	165	this work
*Pontolis magnus*	406	[7]
*Dusignathus santacruzensis*	253	[7]
*Dusignathus seftoni*	282	[7]
*Gomphotaria pugnax*	337	[3]
*Aivukus cedrosensis*	207	[7]
*Protodobenus japonicas*	171	[7]
*Ontocetus emmonsi*	283	[19,33]^a^
*Valenictus chulavistensis*	218	[7]
*Odobenus rosmarus*	300	[30]

^a^Based on mandible length for IRSNB M.168 and USNM 9343, formula from Churchill *et al*. [7].

## 5. Discussion and conclusion

During the late Miocene–Pliocene, odobenids show a marked trend of increasing body size, similar to what is observed in other marine mammals [3,4,7,31]. In odobenids this increase seems to have been possible due to a combination of factors, such as extinction of desmatophocids, increased marine productivity and exploitation of other feeding niches (e.g. benthic feeding) [3,4,7,32]. *Nanodobenus arandai* seems to be the exception to this trend. With a body length estimate of 1.65 m (figure 4 and table 3), it lived during a transitional time when desmatophocids or odobenids were the largest pinniped in any assemblage (figure 4 [3, fig. 7]). Its size was actually closer to that of the early otariid *Pithanotaria starri,* which is known from California and was sympatric with large odobenids such as *Imagotaria downsi* and others (figure 4 and table 3) [3,5]. It is possible that *Nanodobenus* was occupying a niche similar to that of *Pithanotaria*, and was replaced by otariids such as *Thalassoleon mexicanus*, known from the overlying Almejas Formation, which occurs sympatrically with the larger odobenids *Aivukus cedrosensis* and *Dusignathus santacruzensis* (figure 4) [5,15]. Other pinnipeds are known from the Tortugas Formation in the study area [15], and examination of the material housed at LACM confirms the presence of a small odobenid (LACM 60914, tentatively referred to *Nanodobenus*). However, the identity of other pinnipeds from that formation needs to be confirmed by examination of specimens in other institutions, although it is evident that multiple pinniped taxa were present.

*Nanodobenus* has a smaller body size to any other odobenid, and smaller than ancestral length (195 cm) estimated by Churchill *et al*. [7], greatly contrasting with that of coeaval odobenids and even more with the slightly younger *Pontolis magnus* (figure 4 and table 3). Interestingly, this mid–late Miocene dwarfism seems to mirror the pattern seen in the southeastern Pacific with the occurrence of a dwarf seal in assemblages dominated by mid–large phocids [34], and the occurrence of several small phocids in the North Sea and Paratethys [35].

## Supplementary Material

Electronic Supplementary Material

## Data accessibility

Additional data are in the electronic supplementary material. This published work and the nomenclature that it contains have been registered in ZooBank. The LSID for this publication is: zoobank.org:pub:1CBF341E-7B98-475A-84BC-DC609371E897.
